# Olive Leaves as a Potential Phytotherapy in the Treatment of COVID-19 Disease; A Mini-Review

**DOI:** 10.3389/fphar.2022.879118

**Published:** 2022-04-13

**Authors:** Shimaa M. Abdelgawad, Mahmoud A. El Hassab, Mohammed A. S. Abourehab, Eslam B. Elkaeed, Wagdy M. Eldehna

**Affiliations:** ^1^ Pharmacognosy Department, Faculty of Pharmacy, Fayoum University, Fayoum, Egypt; ^2^ Department of Medicinal Chemistry, Faculty of Pharmacy, King Salman International University (KSIU), South Sinai, Egypt; ^3^ Department of Pharmaceutics, Faculty of Pharmacy, Umm Al-Qura University, Makkah, Saudi Arabia; ^4^ Department of Pharmaceutical Sciences, College of Pharmacy, AlMaarefa University, Riyadh, Saudi Arabia; ^5^ School of Biotechnology, Badr University in Cairo, Badr City, Egypt; ^6^ Department of Pharmaceutical Chemistry, Faculty of Pharmacy, Kafrelsheikh University, Kafrelsheikh, Egypt

**Keywords:** olive leaves, phytoconstituents, antiviral, SARS-CoV-2, anti-inflammatory, anti-thrombotic

## Abstract

Beginning from December 2019, widespread COVID-19 has caused huge financial misfortunes and exceptional wellbeing emergencies across the globe. Discovering an effective and safe drug candidate for the treatment of COVID-19 and its associated symptoms became an urgent global demand, especially due to restricted information that has been discharged with respect to vaccine efficacy and safety in humans. Reviewing the recent research, olive leaves were selected as a potential co-therapy supplement for the treatment and improvement of clinical manifestations in COVID-19 patients. Olive leaves were reported to be rich in phenolic compounds such as oleuropein, hydroxytyrosol, verbascoside, apigenin-7-*O*-glucoside, and luteolin-7-*O*-glucoside and also triterpenoids such as maslinic, ursolic, and oleanolic acids that have been reported as anti–SARS-CoV-2 metabolites in recent computational and *in vitro* studies. In addition, olive leaf extract was previously reported in several *in vivo* studies for its anti-inflammatory, analgesic, antipyretic, immunomodulatory, and antithrombotic activities which are of great benefit in the control of associated inflammatory cytokine storm and disseminated intravascular coagulation in COVID-19 patients. In conclusion, the described biological activities of olive leaves alongside their biosafety, availability, and low price make them a potential candidate drug or supplement to control COVID-19 infection and are recommended for clinical investigation.

## Graphical Abstract

Plant photo: https://holotropicherbs.com/product/olive-leaf-olea-european-50-gr/ 
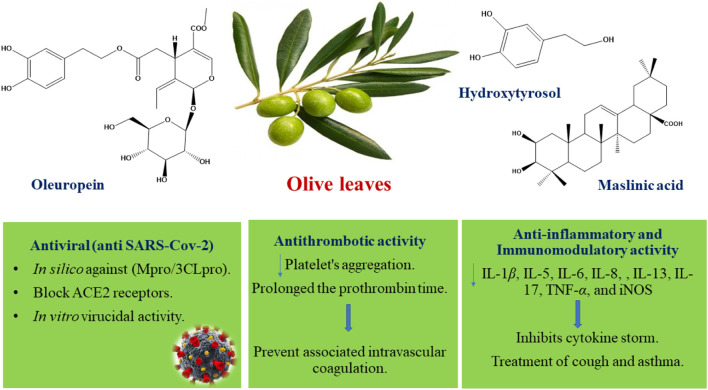

## 1 Introduction

Since December 2019, the COVID-19 pandemic resulted in huge economic deterioration and exceptional uncontrolled health crisis throughout the globe. The total number of COVID-19 cases worldwide until now is about 416,614,051cases and 5,844,097 deaths, as estimated by the WHO, on 17 February 2022 (https://covid19.who.int/). Although several vaccine candidates are now available, only few data have been released regarding the efficacy and safety of vaccines in humans, not to mention that the long-term adequacy of those vaccines still remain as an open address. Nowadays, there is a global trend toward the invention of drug leads that act against COVID-19 infection through different techniques such as *in silico*, *in vitro, in vivo,* and clinical studies of the drug candidates. However, until now, only one drug (Paxlovid^®^) has been approved recently, in December 2022, by the FDA for the treatment and prevention of COVID-19 infection (Parums, 2022); hence, there is a crucial requirement to develop antiviral agents capable of controlling the infection.

WHO reports revealed that COVID-19 disease is most spread in Europe (58%), Americas (23%), Southeast Asia (8%), Western Pacific (7%), Eastern Mediterranean (4%), and then Africa (1%) (O (2022). Weekly epidem, 2022). Additionally, according to the WHO, around 80% of the people in many third-world countries rely on conventional plant sources for their health issues (Ekor, 2014; Ganjhu et al., 2015). Natural products have been historically used for acute respiratory infections, and currently, different natural plant products are being investigated as antiviral agents. (Lin et al., 2014; Ganjhu et al., 2015).

The olive (*Olea europaea* L., family Oleaceae) is a small tree native to Asia, whose cultivation spreads to all the Mediterranean countries, Europe, Iran, and northern Africa (Cimato and Attilio, 2011; Hashmi et al., 2015; Özcan and Matthäus, 2017). Olive trees are abundant and ethnomedically used in the countries where COVID-19 infection is widespread (Hashmi et al., 2015). Olive leaves were reported to exhibit several biological activities such as antioxidant (Benavente-Garcıa et al., 2000; Soni et al., 2006), antihypertensive (Susalit et al., 2011), antihypercholesterolemic (Jemai et al., 2009), cardioprotective (Wang et al., 2008), anti-inflammatory (Khalatbary and Zarrinjoei, 2012), and anti-obesity (Santiago-Mora et al., 2011) activities.

Olive leaf extract was reported to be rich in phenolic compounds such as oleuropein, hydroxytyrosol, verbascoside, apigenin-7-*O*-glucoside, and luteolin-7-*O*-glucoside (Benavente-Garcıa et al., 2000; Goldsmith et al., 2014).

## 2 Phytochemistry Review

Several secondary metabolites with different chemical classification were reported and isolated from olive leaves, such as secoiridoids, flavonoids, triterpenoids, steroids, and lignans.

**FIGURE 1:**
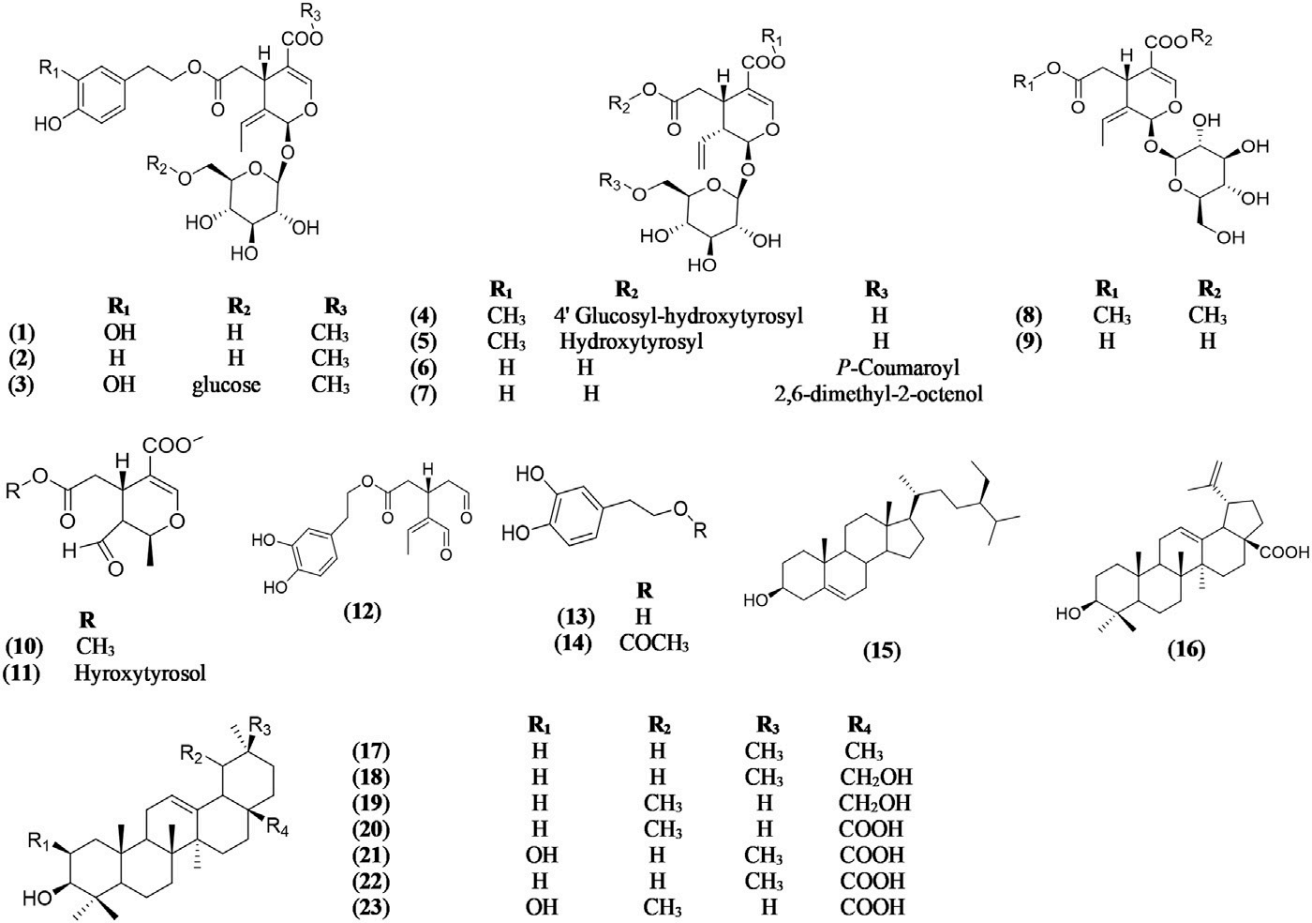
Chemical structures of secoiridoids, triterpenoids, and steroids reported in *Olea europaea* leaves.

Oleuropein (**1**) has been reported from the methanolic and aqueous extracts of leaves of *Olea europaea* (Wichers et al., 2000; Goulas et al., 2009; Hashmi et al., 2015; Nassir et al., 2019). Oleuropein and other secoiridoids such as ligstroside (**2**) (Gariboldi et al., 1986; Hashmi et al., 2015), oleuricine A (**3**), oleuricine B (**4**) (Wang et al., 2009; Hashmi et al., 2015), oleuroside (**5**) (Movsumov, 1994; Duquesnoy et al., 2007; Hashmi et al., 2015), secologanoside, 6′-*E*-p-coumaroyl-secologanoside (comselogoside) (**6**) (Duquesnoy et al., 2007; Hashmi et al., 2015), 6′-*O*-[(2*E*)-2,6-dimethyl-8-hydroxy-2-octenoyloxy]-secologanoside (**7**) (Duquesnoy et al., 2007; Hashmi et al., 2015), oleoside (**8**) (Movsumov, 1994; Duquesnoy et al., 2007; Hashmi et al., 2015), secologanoside (**9**), elenolic acid methyl ester (**10**), hydroxytyrosol-elenolate (**11**) (Gariboldi et al., 1986; Hashmi et al., 2015), and 3,4-DHPEA-EDA (oleacein) (**12**) (Movsumov, 1994; Duquesnoy et al., 2007; Hashmi et al., 2015) were isolated from the extract of leaves of *Olea europaea.* The phenylethanoid precursors of oleuropein; hydroxytyrosol (**13**) and hydroxytyrosol acetate (**14**), were reported in high amount in olive leaves (Goulas et al., 2009) (Figure 1).

The ethyl acetate fraction of *O. europaea* leaves resulted in the isolation of different steroids and triterpenoids such as *β*-sitosterol (**15**) (Mussini et al., 1975; Hashmi et al., 2015), betulinic acid (**16**) (Bianchi et al., 1992; Hashmi et al., 2015), *β*-amyrin (**17**) (Mussini et al., 1975; Wang et al., 2009; Hashmi et al., 2015), erythrodiol (**18**) (Duquesnoy et al., 2007; Hashmi et al., 2015), uvaol (**19**) (Bianchi et al., 1992; Hashmi et al., 2015), ursolic acid (**20**) (Bianchi et al., 1992; Hashmi et al., 2015), maslinic acid (**21**) (Bianchi et al., 1992; Hashmi et al., 2015), oleanolic acid (**22**) (Movsumov, 1994; Duquesnoy et al., 2007; Hashmi et al., 2015), and corosolic acid (**23**) (Bianchi et al., 1992; Hashmi et al., 2015) (Figure 1).

**FIGURE 2:**
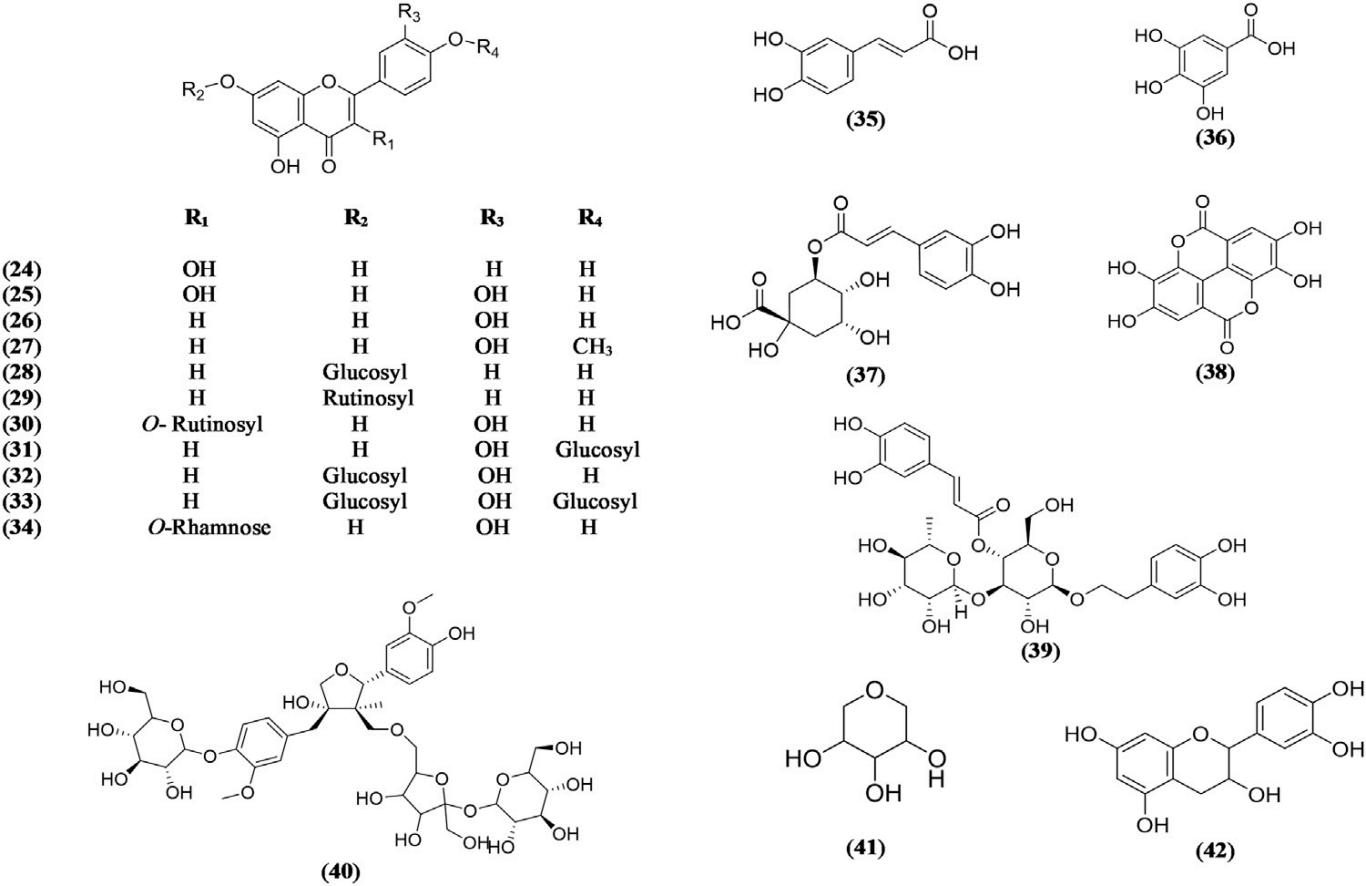
Chemical structures of flavonoids and phenolic compounds reported in *Olea europaea* leaves.

Flavonoids are also important phytochemical constituents reported to be isolated and detected in the extracts of *Olea europaea* leaves. Different types of flavone and flavonols such as aglycones and glycosides were reported in olive leaves such as kaempferol (**24**) (Özcan and Matthäus, 2017), quercetin (**25**) (Özcan and Matthäus, 2017), luteolin (**26**) (Goulas et al., 2009), diosmetin (**27**) (Savournin et al., 2001; Meirinhos et al., 2005; Hashmi et al., 2015), apigenin-7-*O*-glucoside (**28**) (Savournin et al., 2001; Meirinhos et al., 2005; Hashmi et al., 2015), apigenin-7-*O*-rutinoside (**29**) (Bouaziz et al., 2005; Meirinhos et al., 2005; Hashmi et al., 2015), rutin (**30**) (Bouaziz et al., 2005; Meirinhos et al., 2005; Hashmi et al., 2015), luteolin-4′-*O*-glucoside (**31**) (Goulas et al., 2009), luteolin-7-*O*-glucoside (**32**) (Bouaziz et al., 2005; Meirinhos et al., 2005; Goulas et al., 2009; Hashmi et al., 2015), luteolin-7,4′-*O*-diglucoside (**33**) (Savournin et al., 2001; Meirinhos et al., 2005; Hashmi et al., 2015), and quercitrin (**34**) (Özcan and Matthäus, 2017) (Figure 2).

Several phenolic acids were also isolated from *Olea europaea* leaves, such as caffeic acid (**35**), gallic acid (**36**), chlorogenic acid (**37**), ellagic acid (**38**), and verbascoside (**39**) (Özcan and Matthäus, 2017) (Figure 2).

Other miscellaneous compounds were reported from the *Olea europaea* leaves such as 4′-*O*-β-D-glucosyl-9-*O*-(6″-deoxysaccharosyl) olivil (**40**) (Schumacher et al., 2002), 1,5 anhydroxylitol (**41**) (Campeol et al., 2004), and epicatechin (**42**) (Özcan and Matthäus, 2017) (Figure 2).

Several fatty constituents were reported in the hexane extract of *Olea europaea* leaves, including hydrocarbons, tocopherols, triglycerides, waxes, esters, esterols, terpenic alcohols, terpenic dialcohols, and lineal (Guinda et al., 2002).

## 3 Biology Review

### 3.1 Therapeutic Potential of Bioactive Compounds in the Olive Leaves Extract for the Management of COVID-19 Disease

Unlike other coronaviruses that caused several respiratory diseases, SARS-CoV-2 infection is not only a respiratory but also a systematic infection that exhibited severe immune response and resulted in multiorgan dysfunction and finally death (da Rosa Mesquita et al., 2021). The main symptoms of COVID-19 disease are fever, headache, fatigue, malaise, dry cough, and dyspnea with progression to pneumonia, diarrhea, back pain, and loss of smell and taste (da Rosa Mesquita et al., 2021; Zhou et al., 2020; Kabrah et al., 2021; Tawakul et al., 2021). The symptoms vary among patients depending on the viral load and virus strain (da Rosa Mesquita et al., 2021). The recent epidemiological studies detected certain people who are more vulnerable to COVID-19 infection, including older adults and people with serious health problems such as chronic lung disease, asthma, serious heart illness, and immuno-compromised patients (C (2020). Coronavirus D, 2020; Alyami et al., 2020).

The primary reason for most of the deaths occurring in COVID-19 patients is hyper-inflammation due to the associated cytokine storm, especially IL-6, which resulted in acute respiratory distress syndrome (Cron et al., 2021; Zawawi et al., 2021). Moreover, one of the most dangerous complications of COVID-19 is the associated disseminated intravascular coagulation. Numerous reports showed that COVID-19 is related to the increased rate of thrombotic occasions (Goodman, 2020; Klok et al., 2020; Willyard, 2020). Currently, the therapies used for treatment of COVID-19 patients include different categories such as anti–SARS-CoV-2 monoclonal antibodies, antiviral products, immunomodulators, antithrombotic therapy, and supplements (H (2021a). Therapies. A, 2021a). Several medications are prescribed for the management of hospitalized COVID-19 patients such as remdesivir, dexamethasone, tofacitinib, baricitinib, tocilizumab, and sarilumab (H (2021b). Therapeutic, 2021b). Until now, several treatments have been prescribed for COVID-19 patients to relieve symptoms and manage complications, but no single treatment affords antiviral activity and symptomatic treatment.

The total extract of olive leaves and their compounds were reported in several studies for their antiviral, anti-inflammatory, immunomodulatory, and antithrombotic activities as described in this article. In addition, several products in the market that contain standardized olive leaf powder or extract are available for consumers, and most are standardized to 20% or 50% oleuropein. The aim of this study is to emphasize the potential benefit of the natural supplement (olive leaves) to undergo further *in vivo* or clinical investigations.

### 3.2 Antiviral (anti-SARS-CoV-2) Activity of Olive Leaf Compounds

#### 3.2.1 *In Silico* and Computational Antiviral Studies

The antiviral activity of olive leaf metabolites against SARS-CoV-2 was reported in several *in silico* computational studies. Several viral targets were tested, such as viral proteases (Mpro/3CLpro, PLpro), TLRs, ACE2, RBD, NSP15, HSPA5 SBD*β*, TMPRSS2, S protein, and furin (Yu et al., 2020a; Yu et al., 2020b; Derosa et al., 2020; Elfiky, 2020; Hashem, 2020; Hu et al., 2020; Jena et al., 2020; Khaerunnisa et al., 2020; Sampangi-Ramaiah et al., 2020; Shawky et al., 2020; Suručić et al., 2020; Vardhan and Sahoo, 2020; Vijayan and Gourinath, 2020; Khan et al., 2021) (Table 1).

**TABLE 1:** Binding affinity (Kcal/mole) scores of olive leaf compounds against several targets in SARS-CoV-2.

Compound name	Mpro/3CLpro	Plpro	ACE2	RBD	TLRs	NSP15	HSPA5 SBD*β*	TMPRSS2	S protein	Furin	References
Oleuropein	−7.83										Khaerunnisa et al. (2020); Vijayan and Gourinath (2020)
Hydroxytyrosol			−6.54				−5.20	−6.87			Khaerunnisa et al. (2020); Vijayan and Gourinath (2020)
Oleanolic acid	−7.8										Sampangi-Ramaiah et al. (2020)
Maslinic acid			−10.2	−9.3							Vardhan and Sahoo (2020)
Ursolic acid	−8.9										Vardhan and Sahoo (2020)
Rutin	−8.67				−5.29: −9.58						Hu et al. (2020)
Luteolin	−8.2	−7.1	−10.1								Yu et al. (2020a)
Luteolin-7-*O*-glucoside	−8.47										Khaerunnisa et al. (2020)
Quercetin	−6.25	−4.62									Derosa et al. (2020)
Kaempferol	−6.4										Khan et al. (2021)
Verbascoside	−11.721	−14.041									Shawky et al. (2020)
Caffeic acid	−4.387						−6.3				Elfiky (2020); Hashem (2020)

Gallic acid			−5.244						−4.808	−7.486	Suručić et al. (2020)
Chlorogenic acid			Combines with ACE2 Gln42/Asp38 in the form of hydrogen bonds				−6.8				Yu et al. (2020b); Elfiky (2020)
Ellagic acid			−6.854					−6.829	−6.114	−7.801	Suručić et al. (2020); M. Mostafa et al. (2022)
Epicatechin			−8.9						−10.5		Jena et al. (2020)

#### 3.2.2 *In Vitro* Antiviral Studies

Several studies reported the antiviral activities of compounds present in olive leaves against SARS-CoV-2.

A hydroxytyrosol-rich cream (HIDROX^®^) showed virucidal activity against SARS-CoV-2 through structural changes in SARS-CoV-2, which is attributed to changing the molecular weight of the spike proteins and disrupting the viral genome (Takeda et al., 2021). Another study showed that the infection of Vero E6 cells by SARS-CoV-2 was decreased by luteolin with an EC_50_ value of 10.6 μM (CC_50_ = 155 μM) (Yi et al., 2004; Russo et al., 2020). In addition, kaempferol inhibited SARS-CoV-2 replication *in vitro* with % of inhibition equaling 88.33, 93.33, and 40.00% at a concentration of 125.00, 62.50, and 31.25 μM, respectively (Khan et al., 2021).

#### 3.2.3 Clinical Antiviral Studies

A spray containing 3.80% hydroxytyrosol was proven for its activity as protection against SARS-CoV-2 infection in 50 volunteers, and it showed decrease in the viral load and cure in six patients within ten days (Ergoren et al., 2020).

### 3.3 Anti-Inflammatory, Antipyretic, and Analgesic Activities

Olive leaf extract significantly decreased the secreted protein levels of IL-6 and IL-8, and also, mRNA expression of E-selectin in serum amyloid A (SAA)–stimulated human coronary artery endothelial cells (HCAECs) and reduced matrix metalloproteinase (MMP2) levels in unstimulated cells (Burja et al., 2019) (Table 2). In addition, oleuropein was reported as a potential anti-inflammatory molecule for treating asthma and chronic obstructive pulmonary disease (COPD) when administered orally at a dose of 10–20 mg/kg in the experimental BALB/c mice model. It inhibited pulmonary inflammation and subsequent asthmatic fibrosis and alveolar emphysema *in vivo* of asthma induced by exposure to interleukin IL-4, ovalbumin (OVA), or cigarette smoke (CS). The mechanism of action of oleuropein was by reducing the influx of eosinophils and lymphocytes in the airway and diminishing IL-4 secretion in the lung, suppressing the infiltration of macrophages and neutrophils by blocking the induction of intercellular adhesion molecule 1 (ICAM-1), F4/80, CD68, and CD11b in airways (Kim et al., 2018).

**TABLE 2:** Anti-inflammatory, antipyretic, and analgesic activities of olive leaf compounds against SARS-CoV-2.

Active extract or compound	Mechanism of action	References
The ethanolic extract	The olive leaves extract exhibited anti-inflammatory and analgesic activities at doses of 250 and 500 mg/kg through both peripheral and central mechanisms. The extract was active as an analgesic at a level similar to that of indomethacin, and it exhibited antipyretic and anti-inflammatory activities comparable to those of paracetamol.	Laaboudi et al. (2016); Ghazi and Al-Baghdadi (2018)
Oleuropein	• In an *in vitro* study, oleuropein at the dose of 20 μg/ml showed anti-inflammatory activity after 30 min of administration by inhibiting the secretion of tumor necrosis factor alpha (TNF-*α*)–induced matrix metalloproteinase 9 (MMP-9), which additionally resulted in the anti-atherosclerosis effect.	Qabaha et al. (2018); Visioli et al. (2002); de la Puerta et al. (1999); Dell’Agli et al. (2010); Alsharif et al. (2020)
	• *In vivo* administration of oleuropein in a carrageenan-induced pleurisy mouse model significantly reduced the secretion of inflammatory mediators such as TNF-*α* and IL-1*β*.	
	• Oleuropein promoted nitric oxide secretion in LPS-treated macrophages by inducing nitric oxide synthase enzyme, and thus, stimulated the activity of immune competent cells.	
	• Oleuropein additionally inhibited the other inflammatory markers such as leukotriene B4 secretion and lipoxygenase activity.	
Hydroxytyrosol (HXT)	• HXT was found to attenuate the pro-inflammatory markers such as iNOS, COX-2, and TNF-*α* in LPS-challenged human monocytic THP-1 cells *in vitro*.	Gong et al. (2009); Granados‐Principal et al. (2011); Silva et al. (2015)
	• HXT was reported to alleviate the oxidative damages of inflammations by inhibiting the lipoxygenase and cyclooxygenase enzymes of the arachidonic acid pathway.	
	• *In vivo,* HXT had reduced the expressions of pro-inflammatory cytokines such as TNF-*α* and IL-1*β* in inflammatory diseases.	
Terpenoids: Oleanolic acid (OA), Ursolic acid (UA), Maslinic acid (MA), and Uvaol	• OA showed antiallergic and anti-inflammatory effects in the airways by downregulating the infiltration of eosinophil, IL-5, IL-13, IL-17, and TNF-*α* production.	Singh et al. (1992); Raphael and Kuttan (2003); Ikeda et al. (2008); Kashyap et al. (2016); Du et al. (2020); Lee et al. (2020)
	• UA exhibited *in vivo* anti-inflammatory activity in an arthritic balb/c mice model by suppressing pro-inflammatory cytokines such as IL-2, interferon (IFN), and TNF from T helper cell-2 (Th2), inactivating the pro-inflammatory enzyme sPLA2 and suppressing the production of COX-2 and iNOS.	
	• MA demonstrated anti-inflammatory effect by suppressing the expression of NF-ĸB by working against the binding of transcription factor (TF) NF-ĸB to the promoter sequence of COX-2 and iNOS, prevention of NF-ĸB phosphorylation, nuclear translocation, and DNA-binding.	
	• Uvaol exhibited mucolytic and anti-inflammatory activities in the airways by inhibiting the infiltration of eosinophils, downregulating the production of IL-5 and IL-1*β*, and inhibiting the phosphorylation of mitogen-activated protein kinases (ERK1/2).	

### 3.4 Immunomodulatory Activity

Olive leaf extract and its phytoconstituents exhibited immune-modulatory effects by reducing the expression of pro-inflammatory mediators (IL-1*β*, IL-6, IL-8, TNF-*α*, and iNOS) that also resulted in its anti-inflammatory effects (Randon and Attard, 2007; Sánchez-Tena et al., 2013; Vezza et al., 2017; Harun et al., 2020). In an *in vivo* study using the mucosal explant cultures of Crohn’s disease patients and healthy volunteers, the ethanolic extract of olive leaves (0.1–100 μg/ml) reduced the expression of pro-inflammatory mediators such as IL-1*β*, IL-6, IL-8, TNF-*α,* and iNOS and improved the integrity of the epithelial barrier and restored the expression of ZO-1, MUC-2, and TFF-3 (Vezza et al., 2017). Oleuropein was reported to stimulate the proliferation and aggregation of lymphocytes and induce blastogenesis *in vitro* (Randon and Attard, 2007). Erythrodiol strongly inhibited the production of IL-6 (Harun et al., 2020). In addition, both uvaol and oleanolic acid significantly inhibited the production of TNF-*α* at a concentration of 100 μmol/L (Harun et al., 2020). Maslinic acid suppressed the chronic inflammation and exhibited antimodulatory activity comparable with that of dexamethasone through the development and sustainability of intestinal adenomatous polyps in ApcMin/+ (Sánchez-Tena et al., 2013).

### 3.5 Antithrombotic Activity

The ethanolic extract of olive leaves and its phytoconstituents such as hydroxytyrosol, hydroxytyrosol acetate, and maslinic acid reduced the platelet aggregation and exhibited antithrombotic activity by different mechanisms (Elzagallaai et al., 2000; Dub and Dugani, 2013; Vilaplana-Pérez et al., 2014; Kim et al., 2020). In a thromboplastin-induced thrombosis rabbit model, the pretreatment with the olive leaf ethanolic extract (100 or 200 mg/kg per day) for eight weeks modified the extrinsic coagulation pathway and drastically prolonged the prothrombin time (PT) in contrast to the control group. The extract also changed the thrombus morphology; the thrombus was filament-like in the treatment group, while it was thick in the control group and completely occluded the vein (Dub and Dugani, 2013).

Hydroxytyrosol reduced human platelet aggregation through a reduction in thromboxane B2 level, a platelet-aggregating and vasoconstrictor agent, which is the chemically stable and inactive form of thromboxane A2 (Vilaplana-Pérez et al., 2014).

Oral administration of hydroxytyrosol and hydroxytyrosol acetate for seven days in rats inhibited platelet aggregation with similar effectiveness of acetylsalicylic acid by decreasing thromboxane synthesis and increasing vascular nitric oxide (NO) production (Vilaplana-Pérez et al., 2014).

Maslinic acid was reported to regulate platelet aggregation and exhibited antithrombotic activity by different mechanisms. It inhibited protein kinase C (PKC) activation by activating the phosphorylation of myristoylated alanine-rich C kinase substrate (MARCKS), which is a phosphorylated substrate of PKC. Maslinic acid inhibited the enzymatic activity of coagulation factor Xa (FXa) and platelet aggregation induced by adenosine diphosphate (ADP) and a thromboxane A2 (TXA2) analog (Elzagallaai et al., 2000; Kim et al., 2020).

## 4 Conclusion

Olive leaves are rich in bioactive secondary metabolites. The major secoiridoid (oleuropein and its metabolite hydroxytyrosol), triterpenes (such as oleanolic, ursolic, and maslinic acids), and flavonoids (luteolin and kaempferol) exhibited *in silico*, *in vitro*, or *in vivo* antiviral activities against SARS-CoV-2.

Apart from the antiviral properties, these bioactive compounds significantly modulated several signaling pathways and demonstrated various activities such as anti-inflammatory, antipyretic, analgesic, immunomodulatory, and antithrombotic properties. These compounds provide a potential natural source to control the cytokine storms observed during COVID-19 infection, manage the symptoms, and protect against complications.

The potential antiviral activity of olive leaves and their other described benefits such as biosafety, availability, and low cost, along with the absence of an effective treatment for COVID-19 infection, makes olive leaves a potential natural remedy for the treatment and control of COVID-19. Clinical studies should be conducted with this plant and its metabolites to prove its efficacy in the treatment of COVID-19 infection.

## Abbreviations

SAA, serum amyloid A; HCAEC, human coronary artery endothelial cells; MMP2, matrix metalloproteinases; COPD, chronic obstructive pulmonary disease; OVA, ovalbumin; CS, cigarette smoke; ICAM-1, intercellular adhesion molecule 1; TNF-α, tumor necrosis factor alpha; MMP-9, matrix metalloproteinase 9; NO, nitric oxide; HXT, hydroxytyrosol; OA, oleanolic acid; UA, ursolic acid; MA, maslinic acid; COX, cyclooxygenase; IFN, interferon; Th2, T helper cell-2; ERK1/2, mitogen-activated protein kinases; PT, prothrombin time; PKC, protein kinase C; MARCKS, myristoylated alanine-rich C kinase substrate; FXa, coagulation factor Xa
